# An Evaluation of the Organ Dose Received by Cardiologists Arising From Angiography Examinations in Educational Hospital in Rasht

**DOI:** 10.5539/gjhs.v8n7p185

**Published:** 2015-11-18

**Authors:** Akram Shoshtary, Jalil Pirayesh Islamian, Mohsen Asadinezhad, Alireza Sadremomtaz

**Affiliations:** 1 Department of Medical Physics, Faculty of Medicine, Tabriz University of Medical Sciences, Tabriz, Iran; 2 Department of Biophysics and Biochemistry, Faculty of Medicine, Guilan University of Medical Sciences, Guilan, Iran; 3 Department of physics, Guilan University, Guilan, Iran

**Keywords:** coronary angiography, radiation dose, radiation worker, thermo luminescent dosimeter

## Abstract

Interventional procedures, cine acquisitions and operation of fluoroscopic equipment in high-dose fluoroscopic modes, involve long fluoroscopic times which can lead to high staff doses. Also, Coronary angiography (CA) procedures require the cardiologist and assisting personnel to remain close to the patient, which is the main source of scattered radiation. Thus, radiation exposure is a significant concern for radiation workers and it is important to measure the radiation doses received by personnel and evaluate the parameters concerning total radiation burden. In this research, we investigated radiation doses to 10 cardiologists performing 120 CA procedures. Using thermo luminescent dosimeters doses to the wrists, thyroid and eyes per procedure were measured. Based on the measured dose values, maximum doses to the Left wrist, Right wrist, thyroid and eyes of cardiologist were measured 241.45 µSv, 203.17 µSv, 78.21 µSv and 44.58 µSv, respectively. The results of this study indicate that distance from the source, use of protective equipment’s, procedure complexity, equipment performance, and cardiologist experience are the principal exposure-determining variables. It can be conclude that if adequate radiation protection approaches have been implemented, occupational dose levels to cardiologists would be within the regulated acceptable dose limits.

## 1. Introduction

Interventional cardiology(IC) is a branch of cardiology where x- ray guided procedures are performed to diagnose and treat various heart disease which become recently leading the main cause of death (Domienik et al., 2012). IC procedures are performed in ever increasing numbers around the world (Durán et al., 2013). The main reason is that IC permits specialists to avoid complicated invasive surgery, which some patients might not tolerate because of factors such as patient’s age or pathology, and this results in a reduced length of hospital stay in comparison with coronary artery bypass grafting (Baim & Grossman, 1994).

Coronary angiography (CA) is defined as the coronary vessels radiographic visualization after direct opacification with contrast media. It is most commonly used to determine the coronary anatomy, the presence and extent of obstructive coronary artery disease (CAD) and to assess the feasibility and appropriateness of various therapy forms such as revascularization by percutaneous or surgical interventions. Despite the advances in other diagnostic methods, it is still “the golden standard” of coronary disease diagnostics (Caluk, 2011).

CA is a complex combination of relatively low dose screening (fluoroscopy mode; fluoro) and relatively high dose rapid sequence of radiographic exposures recorded in a film (cineradiography mode; cine).

Radiation dose is an unintended consequence of some diagnostic and interventional procedures (Mercuri et al., 2008). Thus, proper dose assessment is a prerequisite for its management. Although interventional cardiac examinations account for 12% of all radiological procedures, they are responsible for delivering the highest radiation dose (up to 50% of the total collective effective dose) (Sun et al., 2013).

Cardiologists encounter much more radiation than most other medical staff due to their working position being close to the beam and the patient (the source of scatter radiation). Therefore, radiation exposure is a significant concern for interventional cardiologists due to the increasing workloads and the procedures complexity over the last decade (Sun et al., 2013).

Dr. Heshmat hospital in Rasht - Iran is one of the important therapeutic centers, where cardiology department accepts many patients for cardiac diagnostic and therapeutic study. On average, about 3600 CA tests are performed annually in this center.

This present survey was focused on estimating cardiologist doses for different anatomical location during CA procedures by thermo luminescent dosimeter (TLD), as the most widely used technology for personal dosimetry (Foti et al., 2008).

## 2. Methods and Materials

All interventional procedures were performed in the catheterization department of the Dr. Heshmat university hospital of Rasht, which equipped with a variety of radiation protection equipment, including: Personal protective equipment (apron, thyroid collar, lead glasses) and room protective equipment (protective drapes suspended from the table and from the ceiling). The IC examinations were performing using a SIMENS system (Axiom Artis dfc model, Germany) with an under couch tube. This system’s features are listed in Table 1:

**Table 1:** Imaging system features

Field size(cm^2^)	13, 17, 21, 23 (cm^2^)
Frame Rate(F/Sec)	10,15 (F/Sec)
Fluoro(kVp)	30-80 (kVp)
Fluoro (mA)	4-15 (mA)
Cine(kVp)	120 (kVp)
Cine(mA)	30-170 (mA)

Field size= radiation field.

The total filtration was automatically varied depending on the selected imaging mode having values between 2 and 3.5 mm Al, and tube setting such as peak voltage and tube current were controlled by the automatic exposure control (AEC).

To verify the timer and tube voltage, Diavolt (PTW-Freiburg), made in Germany, were used. To perform the test, dosimeter was located at the interventional reference point. No differences has been spotted between angiography system timer and dosimetery system. The difference in voltage was less than 3%.

Procedures were performed by residents, visiting cardiologists and trainees.

From June 2014 to December 2014, the samples were randomly selected, including male and female patients undergoing CAG without any optional criteria. Patient’s demographic data (height, age and weight) are mentioned in Table 3. The total number of the patients were 76 male and 44 female ones.

**Table 2:** Cardiologists demographic data

	Sex	Age (Year)	Height (Cm)	Weight (Kg)	Experience (Year)	Specific disease
C1	M	42	181	103	5	No
C2	M	39	179	83	4	No
C3	F	45	173	95	4	No
C4	F	49	159	72	4	No
C5	M	30	165	79	3	No
C6	M	37	172	87	2	No
C7	M	36	184	68	3	No
C8	F	39	176	70	2	No
C9	F	35	165	91	2	No
C10	M	33	157	84	3	No

F: Female; M: Male.

**Table 3:** Patient demographic data

	Height[cm]	weight[kg]	age[years]
Average	157.6	76.90	58
Maximum	178	118	69
Minimum	132	54	47

Dose measurements were performed using thermoluminescent dosimetetrs (TLDs) LiF crystals, doped with magnesium, copper and phosphorus (LiF: Mg, Cu, P). Formerly, the annealed TLDs, in a Harshaw 3500 TLD reader at temperature of 240 C for 10 minutes, were calibrated at the secondary Standard Dosimetry Laboratory (SSDL), by giving them a certain dose of radiation. The calibration was performed with the calibration detectors in a plexi holder and irradiated with a Cs-137 source (with 0.667 MeV gamma rays).

Dosimeters were stuck at six positions during the procedure. The dose received by 10 cardiologists in a single examination was determined by placing thermoluminescent dosimeters in the selected points on the body where apparently higher doses are expected.

Ten Background TLDs were kept for every measurement and the background signal was subtracted from the measured thermoluminescence signals. Every TLD-reading was also corrected for the individual sensitivity of the respective TLD.

While performing the examinations, all the physicians included in the study were wearing lead aprons, thyroid collars, and protective glasses equivalent to 0.5 mm lead. Also, lead glass screens and above and below table shield were used. For each examination, we recorded the measurement protocol. The protocol included the information on the working procedure and the protective measures used, the position of the cardiologist with respect to the X-ray beam, complexity of the procedure, accessing the catheter, the operator’s experience, the radiation field parameters; i.e. tube voltage; DAP value; fluoroscopy time.

The mean fluoroscopy time, the number of procedure and image and mean DAP for patients subjected to CA are presented in Table 4.

**Table 4:** Dosimetric parameters for monitored procedures

Physician	Number of procedure	Mean fluoroscopy time ± SD (min)	Mean DAP ±SD (µGy.m^2^)
C1	12	5.57±1.19	2085.56±1210.05
C2	12	2.74±1.97	2055.07±921.50
C3	12	3.22±3.25	2389.24±1336.76
C4	12	2.75±1.03	2349.53±801.97
C5	12	3.08±2.81	2469.08±739.74
C6	12	3.26±1.93	2856.83±1646.26
C7	12	3.24±2.31	2377.75±1147.88
C8	12	3.32±2.07	2791.96±1403.13
C9	12	4.54±5.01	2953.91±1540.0
C10	12	4.30±506	2638.4±1544.14

The TLDs were placed inside thin plastic bags to be protected from the physical and chemical damage, and taped on the parts of the body were to be monitored. In brief, 6 TLD were used for each test (right and left wrist on the palmar side, under and outside thyroid shield, and under and over the lead glasses). The measurement protocol was common for all procedures so that all results are homogenized and can be analyzed and compared. The TLDs were always read 24 h after irradiation using a Harshaw 3500 TLD reader. The dose per procedure to the wrists, thyroid and eyes was reported in units of µSv.

Finally, the dose reduction (%) of thyroid and eye was determined by the following equation:


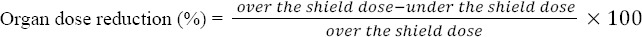


The data are summarized and reported with Meam, SD, Min and Max dose indicators. Also the data were analyzed using one- way ANOVA and SPSS22 software. The significance level was considered p<0.05.

## 3. Results

**Figure 1:**
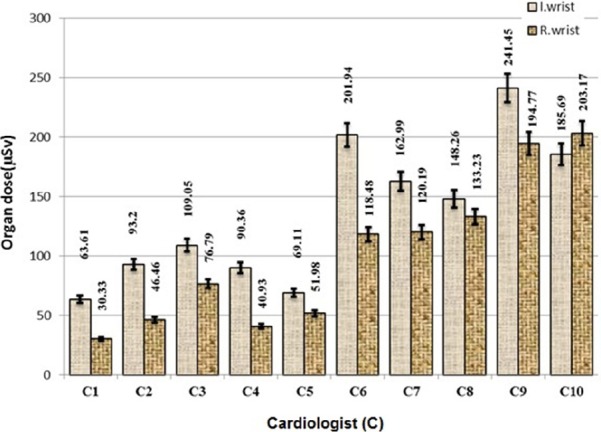
The average dose in each right and left wrists of 10 cardiologists (C1-C10) from interventional cardiology examinations

Figure 1 shows the results on the average dose in each right and left wrists of 10 cardiologists.

Depending on the type of procedure and the technique used, the operator dose per procedure, obtained from 63.61 to 241.45 µSv at the Left wrist and 30.33 to 203.17 at the Right wrist, and highest doses are concerned to 9^th^ cardiologist’s Left wrist and 10^th^ cardiologist’s Right wrist.

It was also observed that for most cardiologists, the Left wrist dose was more than Right one because of proximity to the tube. No significant differences was observed in dose value of Left and Right wrist of C1 –C5 cardiologists.

**Figure 2:**
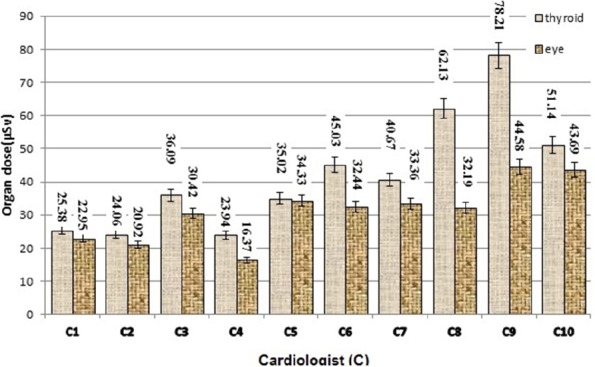
The measured thyroid and eyes dose of 10 cardiologists (C1-C10) per procedure from interventional cardiology examinations

The measured thyroid and eyes dose of 10 cardiologists per procedure from CA examinations is shown in Figure 2.

According to the obtained data, presented in Figure 2, the maximum thyroid and eye doses were found 78.21µSv and 44.58 µSv, respectively that were related to 9^th^ cardiologist (C9). Table 5 points out the summary of cardiologists’ data for CA procedures in the present study.

**Table 5:** Summary of cardiologists’ data for CA procedures in the present study

Organ	Mean	S.D	Med	Min	Max
Left wrist	130.9	88.83	106.94	30.55	376.01
Right wrist	93.65	82.01	57.21	19.72	385.91
Thyroid	40.05	21.65	32.36	11.04	101.00
Eye	30.15	15.01	26.67	10.16	95.01

Table 6 shows organ dose reduction when using protective shields.

**Table 6:** Thyroid and eye dose reduction (%)

	Thyroid dose reduction (%)	Eye dose reduction (%)
C1	36	34
C2	35	35
C3	27	27
C4	38	34
C5	33	26
C6	39	37
C7	53	38
C8	28	25
C9	41	24
C10	41	30

## 4. Discussion

For CA examinations, the obtained results have been analyzed statistically and no significant differences has was seen between the results at the confidence level 95%.

The present results show an ample range of variation in doses at the locations monitored on the physicians, especially at their wrists, confirming the influence of equipment features, the complexity of the procedure, fluoroscopy time, work technique, shielding, and perhaps the most important of all, the cardiologist’s experience. Some of the factors influence the dose to cardiologists, are listed in Table 7.

**Table 7:** Some of the factors affecting cardiologists dose in CA.

Category	
**Patient**	Clinical problem, lesion characteristic, body habitus
**Physician**	Body size and height, experience and skill, work load, procedural technique, position with respect to patient
**Shielding**	Equipment- mounted shielding and personal protective shields
**Angiographic equipment**	

Examination time is an important factor, which related to experience and skill of cardiologists, but no meaningful correlation was found between the examination time and dose to cardiologist.

The mean DAP values of this survey for CA procedures are comparable with mean DAP from other studies.

As illustrated in Table 2, mean DAP in the present research is less than other studies.

This variations in DAP values for similar procedures could arise from changes in field size, cine and fluoroscopic time, physician experience and skill and the complexity of cases.

There is a great difficulty in comparing the dosimetric results in the various studies because many different factors influence the cardiologist’s doses. Generally, the factors influencing the dose were grouped in: the factors related to the patients (age, sex, weight, etc.), the ones related to the equipment and the factors related to cardiologist (technique, screening time, number of procedures, type of the procedures, the operator’s skill, the training level in radiation protection, etc.) (Jokic et al., 2011). Table 9 presents a comparison of our data with the similar studies:

**Table 8:** Comparison of this study with literature in CA

	Mean DAP value [Gy.cm^2^]
Vañó et al (1995)	66.5
Padovani et al (1998)	55.9
Van de putte et al (2000)	60.6
Karambatsakidou et al (2005)	49.0
This study	25.23

**Table 9:** The data on the organ dose (in µSv) of radiation workers for interventional cardiology in three studies

Organ	Present study (CA)	Efstathopoulos et al (2011) (IC)	Zare et al (2007) (CA)
Left wrist	63.61-241.45	20-3684	31.8-358.7
Right wrist	30.33-203.17	14-138	12.0-124.0
Thyroid	23.94-78.21	-	1.2-100.3
Eyes	16.37-44.58	0-61	-

The present study showed significant variations in the levels of radiation dose received by cardiologists in different studies. The only study conducted in Mashhad by Zare et al (2007). Suggested the highest dose in right hand, left hand and thyroid 124, 358.7, 100.3 µSv, respectively. Comparing the values obtained in current and previous research indicated that the maximum left wrist and thyroid dose in our study was lower, while the maximum right wrist dose was higher.

As mentioned before, some of the factors responsible for dose variability are the complexity of the procedure, operator’s experience, training level in radiation protection, and type and performance of x-ray equipment available in the catheterization laboratory (Pantos et al., 2009). Our study also indicate the effectiveness of the related parameters on the dose.

Of all CA laboratory personnel, the performing physician is subject to the highest radiation dose, and numerous patients, physician and shielding factors influence the cardiologist’s dose to different degrees.

Chida et al (2013) compared the annual occupational dose among interventional radiology staff. The annual occupational doses of physicians, nurses, and radiologic technologists were recorded. They found that the annual occupational dose for interventional radiology staff was in the order of physicians > nurses > radiologic technologists. This is readily explained by the relative proximity of the cardiologist to the patient and the x-ray beam. Also, Kim and Miller (2009), reported that decreasing the distance from 1m to 0.75m doubles the occupational radiation dose.

An interesting study was performed by Silva et al (2011) evaluating the values of radiation doses received by physician during IC procedures. The mean effective dose per procedure for physician was 11µSv, and the highest mean equivalent doses were 382 in the left hand and 150µSv in the left eye.

Another study was performed by Vano et al., (2006) that evaluated the cardiologist’s occupational doses for a period of 15 years (1989 to 2004). The mean values in mSv/year decreased from 11.6(1989-1992) to 1.6(1993-1998) and to 1.2(1999-2004).

Efstathopoulos et al (2011), attempted to estimate occupational doses to the extremities and the eyes for interventionists and the assisting laboratory nurse in interventional procedures. For the physicians, the parts of the body that received the highest doses were found at the left wrist, and the annual limits eyes and extremities are not exceeded even for the busiest physicians. Our study also confirmed the results obtained by Efstathopoulos et al (2011).

Unlike their study, we investigated the effect of shielding on dose, which results presented in Table 6.

Dendy (2008) suggested that doses to hands are highly dependent on their position relative to the beam and different hand movements in different procedures.

Domienik et al (2012), analyzed the dose distributions at the region of eye lens and extremities in IC procedures with TL dosimeters (MCP-N). The main objective was to obtain the typical locations of highest doses and to estimate the dose ranges for the selected types of procedures with regard to the following parameters: the X-ray tube geometry, the presence of additional protective equipment and staff position (related to the access). They found that even for the same type of procedure, the variation in the dose levels might be significant depending on the above-mentioned parameters. Therefore, they concluded that the eye dosimeter should be placed aside the eye which is nearest to the X-ray tube. Another study also visualised the dose distribution inside the head, both when protective eyewear were used and without such protection, and they proved that positioning the dosimeter at the eyebrow could lead to an underestimation of the lens dose of as much as 45% (Geber et al., 2011).

Comparison of these findings, the best position for monitoring of eye doses, we found to be at the side of the head nearest to the radiation source, to avoid eyes dose underestimation.

There are a large number of influencing factors that can give a significant variability for the eye doses: different geometries of the various X-ray systems, protective equipment, the complexity of the CA procedures (fluoroscopy time, number of acquired images), work technique (X-ray tube configuration, projections used, etc.) and physician’s experience. In this study, cardiologist C9 has a longer fluoroscopic time and higher DAP value with a slow working behavior, compared to the others. This difference is largely due to his less experience. So it is particularly important that cardiologists are trained in clinical technique and radiation protection.

The eye dose results for the cardiologists collated in this survey show that the operator’s position and body height have major impact on the amount of radiation doses to different parts of the body. As expected, tall cardiologists receive a lower eye dose than a short cardiologists since the distances from the eyes to patient entrance site can significantly vary depending upon the operator’s height.

The results of this study indicated that radiation doses were higher on the left side of the operator’s body (specially the left wrist), because the left side is closer to the X-ray beam when the cardiologist is standing at the patient’s right side, but in the previous study was found that left hand receive the two- fold dose as compared with the right hand during cardiac catheterization (Kantonsspital, 2005). It is important to mention that the radiation dose to the personnel is directly related to the dose to the patient since the major contribution is scattered radiation from the patient (Axelsson, 2007). In addition, cardiologist’s doses can considerably increase if inappropriate X-ray equipment or inadequate personal protection is used (Valentine, 2000).

Radiation shielding is one of the most efficient and easiest methods to protect the staff during interventional cardiology procedures (Todorovic et al., 2011). Fetterly et al., recommended that radiation shields must be thoughtfully placed and actively managed both before and during the procedure to be effective in providing substantial protection during IC procedures (Sun et al., 2013).

As shown in Table 9, there is substantial variation in doses observed for the same type of procedure, indicating that radiological protection practices can be improved. The results of the simulations that were performed by Koukorava et al., suggested that a lead glass (if properly worn) can reduce the doses to the eye lens by a factor 3 to 7 (Vanhavere et al., 2011). Combining various types of shielding (i.e., table-suspended drapes, ceiling-suspended screens, aprons, leaded glasses, mobile shields, and disposable drapes) results in a dramatic dose reduction for the operator (Miller et al., 2009).

It is proved that, leaded glasses decrease the dose to the operator’s eye exposure by a factor of approximately five to 10 (Thornton et al., 2010). Garments, lead goggles, ceiling suspended shields, curtains under the table and other protective equipment provide a significant reduction in occupational doses (Vano, 2003). Of course, the amount of radiation attenuated by a material depends on the elemental composition of the material, its thickness and the energy of the radiation passing through (Francis et al., 2011). In our study, the protection by shielding was also considered by the staff and thus we haven’t considered a comparison for the related doses.

## 5. Conclusion

The interventional cardiologists encounter much more radiation than most other medical staff due to their working position being close to the X-ray beam and the patient (the source of scatter radiation). The increased workload, the complexity of the interventional procedures, fluoroscopy time, and distance from the scattering area on the patient contribute significantly to the amount of radiation exposure to the cardiologists. Training of the operator in radiation protection methods may be a successful action to reduce occupational doses. In order to keep doses as low as applicable, evaluation and follow up of radiation doses received by the physicians should be considered an important part of quality assurance programs for interventional cardiology procedures, and protective devices must be used appropriately.
